# Superoxide dismutase is upregulated in *Staphylococcus aureus *following protoporphyrin-mediated photodynamic inactivation and does not directly influence the response to photodynamic treatment

**DOI:** 10.1186/1471-2180-10-323

**Published:** 2010-12-17

**Authors:** Joanna Nakonieczna, Ewelina Michta, Magda Rybicka, Mariusz Grinholc, Anna Gwizdek-Wiśniewska, Krzysztof P Bielawski

**Affiliations:** 1 Intercollegiate Faculty of Biotechnology University of Gdansk and Medical University of Gdansk, Kladki 24, 80-822 Gdansk, Poland

## Abstract

**Background:**

*Staphylococcus aureus*, a major human pathogen causes a wide range of disease syndromes. The most dangerous are methicillin-resistant *S. aureus *(MRSA) strains, resistant not only to all β-lactam antibiotics but also to other antimicrobials. An alarming increase in antibiotic resistance spreading among pathogenic bacteria inclines to search for alternative therapeutic options, for which resistance can not be developed easily. Among others, photodynamic inactivation (PDI) of *S. aureus *is a promising option. Photodynamic inactivation is based on a concept that a non toxic chemical, called a photosensitizer upon excitation with light of an appropriate wavelength is activated. As a consequence singlet oxygen and other reactive oxygen species (e.g. superoxide anion) are produced, which are responsible for the cytotoxic effect towards bacterial cells. As strain-dependence in photodynamic inactivation of *S. aureus *was observed, determination of the molecular marker(s) underlying the mechanism of the bacterial response to PDI treatment would be of great clinical importance. We examined the role of superoxide dismutases (Sod) in photodynamic inactivation of *S. aureus *as enzymes responsible for oxidative stress resistance.

**Results:**

The effectiveness of photodynamic inactivation towards *S. aureus *and its Sod isogenic mutants deprived of either of the two superoxide dismutase activities, namely SodA or SodM or both of them showed similar results, regardless of the Sod status in TSB medium. On the contrary, in the CL medium (without Mn^++ ^ions) the double SodAM mutant was highly susceptible to photodynamic inactivation. Among 8 clinical isolates of *S. aureus *analyzed (4 MRSA and 4 MSSA), strains highly resistant and strains highly vulnerable to photodynamic inactivation were noticed. We observed that Sod activity as well as *sodA *and *sodM *transcript level increases after protoporphyrin IX-based photodynamic treatment but only in PDI-sensitive strains.

**Conclusions:**

We confirmed that porphyrin-based photokilling efficacy is a strain-dependent phenomenon. We showed that oxidative stress sensitivity caused by the lack of both Sod enzymes can be relieved in the presence of Mn ions and partially in the presence of Fe ions. The fact that Sod activity increase is observed only in PDI-susceptible cells emphasizes that this is probably not a direct factor affecting *S. aureus *vulnerability to porphyrin-based PDI.

## Background

*Staphylococcus aureus*, a major human pathogen causes a wide range of disease syndromes, including life-threatening endocarditis, meningitidis and pneumonia. According to the Centers for Disease Control and Prevention this bacterium has been reported to be the most significant cause of serious infections in the United States [1]. *S. aureus *is able to cause and develop an infection very efficiently due to its ability to produce a few dozen of virulence factors, on one hand, and an ease of antibiotic resistance development, on the other. The most dangerous are methicillin-resistant *S. aureus *(MRSA) strains, constituting 50% of hospital-aquired isolates as well as emerging vancomycin-resistant variants, isolated from some hospital settings [2].

Among several virulence factors, *S. aureus *produces enzymes responsible for resistance against oxidative stress, like catalase and superoxide dismutase (Sod). Sod converts superoxide anion (O_2_·^-^) into hydrogen peroxide (H_2_O_2_), a less potent biological oxidant, which is further decomposed by catalase to water and ground state oxygen. Sod enzyme is produced in response to the presence of reactive oxygen species (ROS) generated endogenously as an effect of oxygen metabolism or, exogenously produced by neutrophils and macrophages. Superoxide anion, which is the product of oxygen reduction, reacts with hydrogen peroxide within the bacterial cell and produces free hydroxyl radical (^.^OH), the most dangerous oxygen species able to interact with virtually any organic substance in the cell. Superoxide anion can reduce hypochlorus acid (HOCl) arose as a result of H_2_O_2 _interaction with phagocyte-derived peroxidases, and further form ^.^OH [3].

The classification of Sod enzymes is based on the type of transition metal present in their active center, including manganese (Mn), iron (Fe), copper (Cu) and a few years ago a nickel (Ni)-containing Sod was described, originally isolated from the cytoplasm of *Streptomyces seoulensis *[4,5]. In the *Escherichia coli *bacterium model, the presence of three Sods were described: Fe- and Mn- Sods localized in the cytoplasm, whereas in the periplasm copper-zinc (Cu-Zn) SOD was detected [6]. *S. aureus *produces three Sod enzymes, encoded by two genes, *sodA *and *sodM *[7,8]. The particular subunits form two kinds of Sod homodimers, i.e. SodA-SodA and SodM-SodM as well as SodA-SodM heterodimers, easily distinguishable on native gels stained for Sod activity [8]. Both, SodA and SodM subunits are believed to possess Mn ions as a cofactor in the active site. Manganese is now believed to play a crucial role in a variety of cellular processes including stress responses [9]. In a range of bacterial pathogens, Mn is recognized as having a major effect on virulence [10,11]. Apart from participating in several enzyme functions, Mn complexes with phosphate and lactate were demonstrated to scavenge ROS [12].

The role of Sod in the pathogenesis of many bacteria was proved. In *S. aureus *however, the results are not unambiguous. The very first analyses of antioxidant enzymes and staphylococcal virulence showed no correlation [13]. Similarly, in a mouse abscess model resulting from *S. aureus *infection, inactivation of *sodA *gene, recognized as the main Sod activity in *S. aureus*, had no impact on staphylococcal virulence [7]. Moreover, mouse kidney infection was not attenuated after *sodM *gene inactivation [14]. On the other hand, examination of a range of virulent versus non-virulent *S. aureus *clinical isolates, showed statistically significant higher Sod activity in the first group studied [15]. Karavolos *et al*. tested the role of Sod in a mouse subcutaneous model of infection and claimed that mutants deprived of either SodA, SodM or both activities had significantly reduced virulence compared to *S. aureus *wild-type SH1000 strain [16].

As bacteria replicate very quickly, the possibility of mutant selection which effectively deals with antibiotic treatment rises. An alarming increase in antibiotic resistance spreading among pathogenic bacteria inclines to search for alternative therapeutic options, for which resistance cannot be developed easily. One such option is photodynamic inactivation of bacteria (PDI). This method involves the use of non toxic dyes, so called photosensitizers (PS), which become excited upon visible light of an appropriate wavelength and eventually a number of ROS are formed [17]. As a consequence of ROS action, which are known to cause severe damage to DNA, RNA, proteins, and lipids, bacterial cells die. Two oxidative mechanisms can occur after light activation of a photosensitizer. When the photosensitizer interacts with a biomolecule, free radicals (type I mechanism), and/or singlet molecular oxygen (^1^O_2_) (type II mechanism) are produced, which are responsible for cell inactivation [18]. In the case of porphyrin-based photosensitizers, ^1^O_2 _seems to be the main ROS generated upon photoexcitation, although O_2_^.^^-^, ^.^OH are also implicated [19]. In a very elegant study by Hoebeke et al., the photochemical action of bacteriochlorin a, a structural analog of protoporphyrin IX, was also demonstrated to be based on both, type I and type II mechanism of action in a 1:1 proportion [20]. Several lines of evidence indicate the effectiveness of PDI *in vitro *against both Gram-positive and -negative species [21,22]. It was also demonstrated that photodynamic inactivation may be applied to inactivate bacterial virulence factors, which represents an advantage over topical antibiotic treatments [23].

In our previous reports we observed that the *S. aureus *response to PDI is strain-dependent. Among clinical isolates some were killed in 99,999%, whereas others in only about 20% in protoporphyrin-based PDI [24]. To understand if the antioxidant enzyme status may be involved in the *S. aureus *response to PDI, we checked the survival rate of the isogenic *sod *mutants of *S. aureus *and compared the activities of Sods in response to PDI on the protein as well as gene expression level.

## Results

### PDI effectiveness towards wild type *Staphylococcus aureus *and its *sod *isogenic mutants

**Figure 1:**
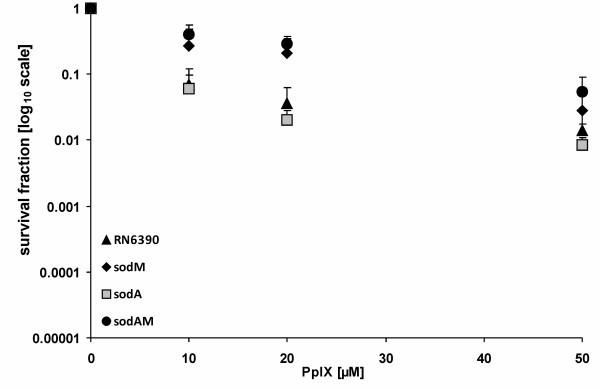
**Protoporphyrin IX-mediated PDI against reference strains in TSB medium**. The bacterial suspensions were illuminated after dark incubation for 30 min. at 37°C with different concentrations of PpIX (up to 50 μM). PDI was tested against reference strains of *S. aureus*: RN6390, RN6390*sodA*, RN6390*sodM*, RN6390*sodAM*. Bacteria were illuminated with 12 J/cm2 624 ± 18 nm light, and survival fractions were determined as described in Methods. Values are means of at least three separate experiments.

With the use of type I or type II oxidative stress quenching agents, we checked that PpIX-mediated PDI is involved in the type I mechanism of oxidative stress induction (production of free radicals) (data not shown). This gave us a rationale to study the influence of Sod on the PDI outcome. In order to check superoxide dismutases' role in photodynamic inactivation we first of all checked whether *S. aureus *RN6390 strain deprived of either SodA, SodM or both of the activities differentially responded to photodynamic inactivation. In our study we used protoporphyrin IX (PpIX) as a photosensitizer. Treatment of *S. aureus *RN6390 and its isogenic *sod *mutants with 0-50 μM PpIX and an irradiation dose of 12 J/cm^2 ^resulted in a weak response to PDI in TSB medium. Wild-type RN6390 showed 1.85 log_10 _units survival reduction in comparison to non PDI-treated cells. In the case of the single SodA and SodM mutants the survival rate accounted for 2.0 log_10 _units reduction and 1.55 log_10 _units reduction, respectively (Figure 1). The double SodAM mutant reduced its survival rate by only 1.3 log_10 _units. Statistical analysis performed on six independent sets of measurements revealed no correlation between the Sod status and PDI response, at least in TSB medium. The observed phototoxic effect was in each case PpIX-concentration dependent in a range of 0-50 μM. We chose one light dose of 12 J/cm^2 ^in all experiments concerning killing data based on our previously published results [24,25].

### Effect of divalent ions on PDI effectiveness towards wild type RN6390 and its *sod *isogenic mutants

**Figure 2:**
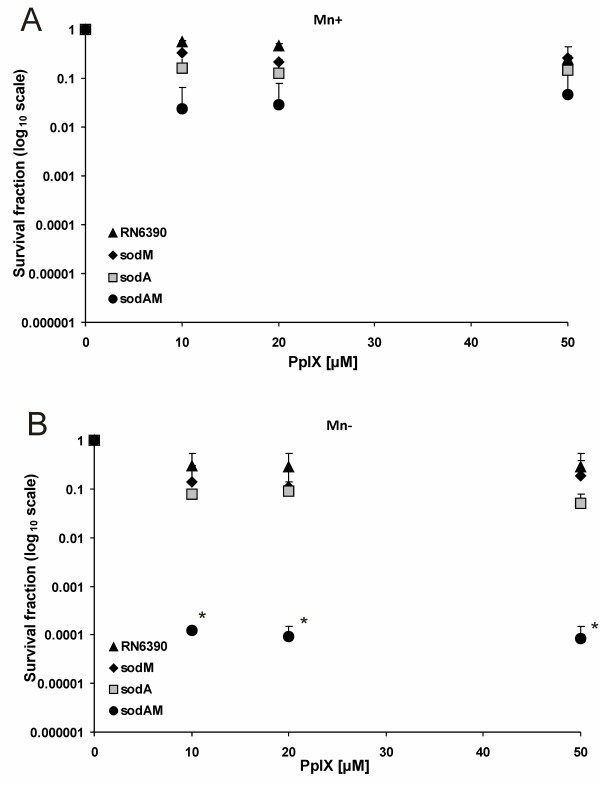
**Mn ions influence on protoporphyrin IX-mediated PDI against reference strains**. The bacterial suspensions were illuminated after dark incubation for 30 min. at 37°C with different concentrations of PpIX (up to 50 μM). PDI was tested against reference strains of *S. aureus*: RN6390, RN6390*sodA*, RN6390*sodM*, RN6390*sodAM *in Mn-supplemented medium (A) and Mn-depleted medium (B). Bacteria were illuminated with 12 J/cm^2 ^624 ± 18 nm light, and survival fractions were determined as described in Methods. Values are means of three separate experiments, and bars are SD. * indicates statistically significant difference in survival drop between RN6390*sodAM *and each of the following strains RN6390, RN6390*sodA*, RN6390*sodM *at each tested concentration (p < 0.05).

As *S. aureus *Sod enzymes are recognized as Mn-containing proteins, we further checked the influence of Mn ion depletion on PDI effectiveness. After cells were cultured in a chemically defined CL medium with and without 20 μM MnSO_4_, PDI procedure was performed according to the Methods section, similarly as with TSB medium. When the CL medium was supplemented with Mn ions the survival rate of *S. aureus *RN6390 and s*odA*, s*odM*, s*odAM *mutants was in general higher in comparison to non Mn-supplemented medium. The values ranged between 0.5 log_10 _units reduction for wild-type RN6390, through 0.6 and 0.9 log_10 _units for the two single *sodM *and *sodA *mutants, respectively, to 1.3 log_10 _units reduction observed in the case of the double *sodAM *mutant (Figure 2A). When the PDI studies were performed in the absence of Mn ions, the survival rate of the three analyzed mutants, but not the wild-type RN6390, decreased. In the case of the *sodM *mutant we observed a 0.9 log_10 _unit reduction in survival rate and 1.3 log_10 _unit reduction when the *sodA S. aureus *was analyzed. For those differences, however, no statistical relevance was proved. Significant difference was observed for the double mutant, whose survival rate dropped by 4.1 log_10 _units (Figure 2B). This result was statistically confirmed. The obtained results suggest that a single Sod activity is sufficient to combat oxidative stress conditions resulting from PDI, whereas *S. aureus *cells without any Sod activity can be rescued by the presence of Mn^++ ^ions. Based on the presented results it can be assumed that oxidative stress sensitivity caused by the lack of both Sod enzymes can be overcame in the presence of Mn ions.

In order to check whether other divalent ions are able to cause such an effect we performed analogous experiments with 20 μM FeSO_4_. Supplementation of CL medium with iron ions resulted in partial restoration of oxidative stress resistance but only in *sodAM *mutant, where the drop in survival rate increased from 4.1 log_10 _units to 2.4 log_10 _units, respectively in CL medium without and supplemented with divalent metal ions (Additional file 1).

### PDI effectiveness towards clinical *Staphylococcus aureus *isolates

**Figure 3:**
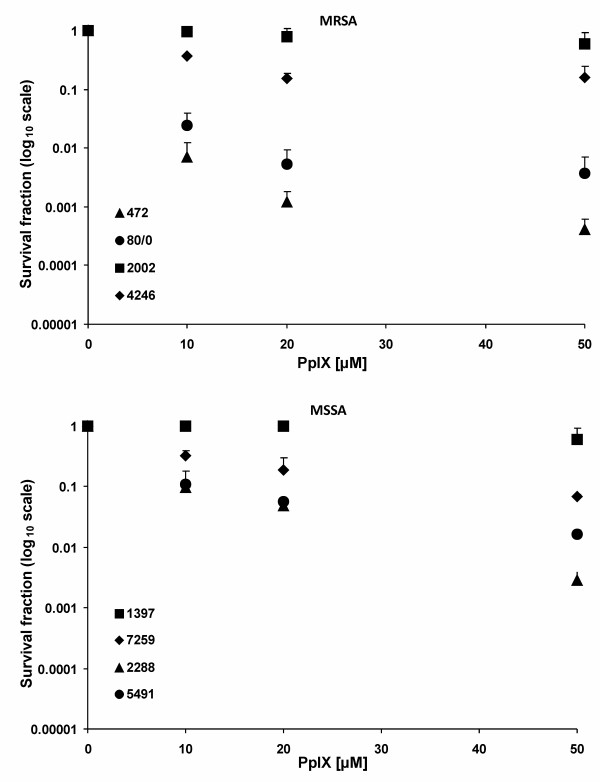
**Protoporphyrin IX-mediated PDI against clinical strains**. The bacterial suspensions were illuminated after dark incubation for 30 min. at 37°C with different concentrations of PpIX (up to 50 μM). PDI was tested against clinical *S. aureus *strains: MRSA, MSSA. Bacteria were illuminated with 12 J/cm^2 ^624 ± 18 nm light, and survival fractions were determined as described in Methods. Values are means of three separate experiments, and bars are SD.

**Figure 4:**
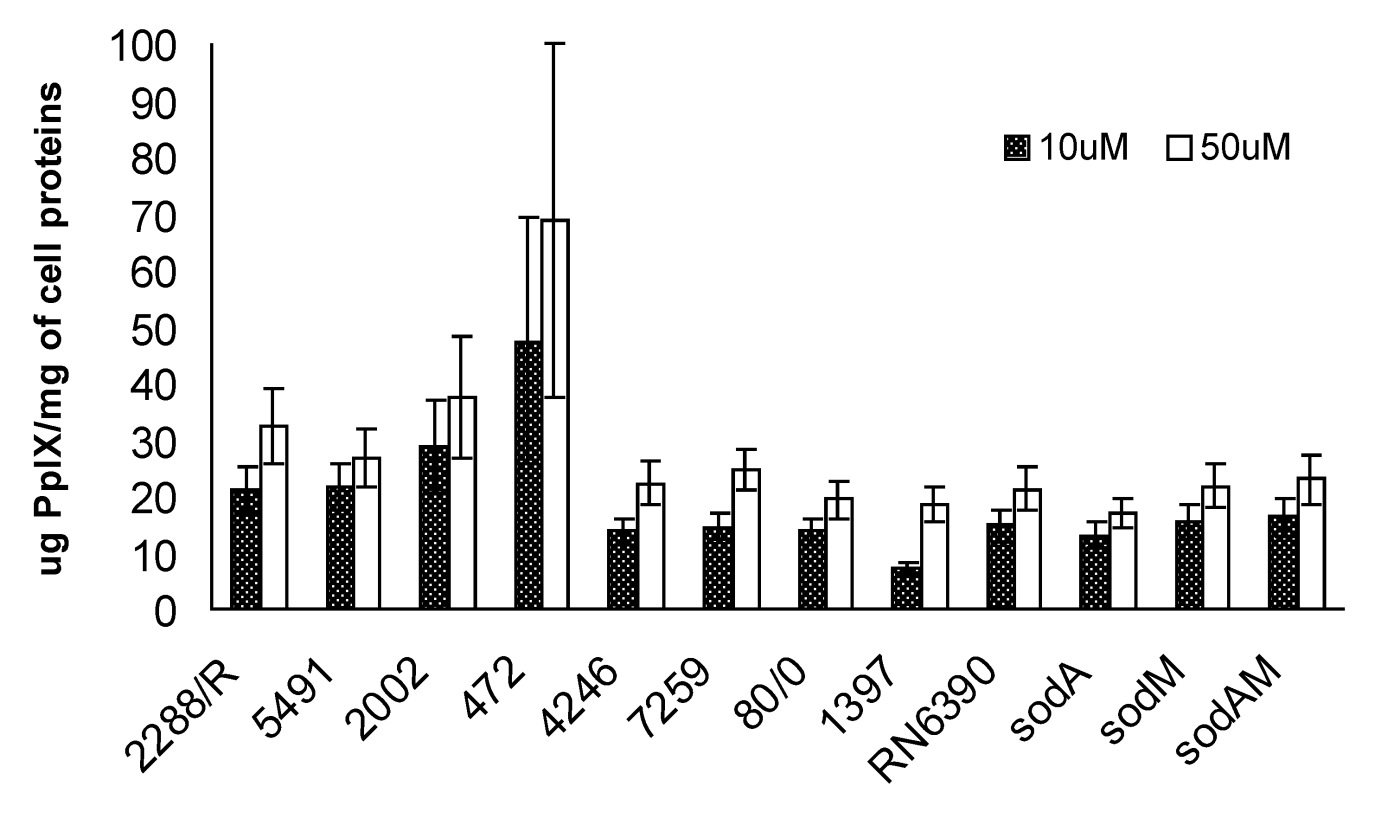
**Uptake of PpIX in the reference and clinical isolates of *Staphylococcus aureus***. Uptake of PpIX (μg/mg cell protein) by *S. aureus *clinical isolates and reference strains. Beneath, the names clinical strains, the name of the parental strain and its *sod *isogenic mutants are indicated. Concentration of PS was 10 μM and 50 μM. PS was incubated for 30 min., washed, dissolved in 0.1 M NaOH-1% SDS, and fluorescence measured as described in the text. Values are means of three separate determinations, and bars are SD.

In order to check PpIX-based PDI effectiveness towards *S. aureus *strains isolated from patients, we chose 4 strains characterized as methicillin resistant (MRSA) and 4 methicillin susceptible strains (MSSA). Examination of the survival rate of the chosen strains resulted in an observation that the response to PDI treatment is strain-dependent. In both groups, MRSA and MSSA, we observed strains which were killed very inefficiently (Figure 3), eg. strains 1397 and 2002 reduced their survival rate only by 0.2 log_10 _units. On the contrary, independent of the methicillin-resistance status we observed strains highly susceptible to PpIX-based photokilling, eg. strains 472, 80/0 and 2288, which reduced their survival rate by 3.4 log_10 _units, 2.4 log_10 _units and 2.5 log_10 _units, respectively. One-way analysis of variance test of the survival of the studied clinical isolates (at 50 μM PpIX concentration) showed statistically significant differences (F = 88,3 p < 0.05). Based on the Tukey post-hoc test, a decrease in the survival of the 4246 strain did not differ from the strains 7259, 2002 and 1397, and further those strains were classified as one group. This group was considered by us as PDI-resistant with the survival decrease not exceeding 1.5 log_10 _units. The next four bacterial isolates (5491, 2288, 80/0, 472) were recognized as PDI-sensitive with the survival decrease of more than 1.5 log_10 _units. It is believed that the effectiveness of PDI depends on the ability of cells to uptake the photosensitizer. We checked whether there are any differences among *S. aureus *strains in PpIX uptake into the cell. Protoporphirin IX uptake in the tested strains did not show much differentiation. It is worth mentioning, however, that in the case of the most PDI-vulnerable 472 strain, PpIX uptake value was 47.4 μg/mg and on the contrary, only 7.3 μg/mg in the case of the most resistant 1397 strain. We observed no apparent correlation between PS uptake and PDI effectiveness. In the case of RN6390 and its isogenic *sod *mutants the uptake was very balanced and ranged between 13.1 and 16.2 μg/mg for the wild type and the mutants (Figure 4).

### Sod activity increases after PDI

In order to assess the amount of Sod activity in strain-dependent response to PpIX-based photodynamic treatment, we measured total Sod activity in *S. aureus *isolates before and after PDI treatment. The activity was measured with the NBT reduction method and was expressed as Sod units per mg of total cell protein content (Methods section). Firstly, the basic level of Sod activity was estimated. Among 8 clinical isolates tested Sod activity was similar and ranged between 1495 U/mg and 2234 U/mg, with the exception of 2288 strain, where the observed activity was the highest and amounted to 3597 U/mg. However, when mean activity values were normalized with respect to the number of c.f.u. (colony forming units), they slightly differed for PDI-susceptible and PDI-resistant strains (23.6 ± 4 U/mg and 33.2 ± 15 U/mg, respectively) (Table 1). These differences appeared much greater when bacterial cells were exposed to PDI. After photosensitization with 50 μM PpIX and illumination with 12 J/cm^2 ^red light, the total Sod activity raised to the mean value of 100.9 ± 30 U/mg in the case of PDI-susceptible strains, whereas only a minor increase in the Sod activity level was observed in PDI-resistant strains (37.1 ± 7 U/mg). This indicates that oxidative stress generated in our experimental conditions greatly induced Sod activity in PDI-susceptible strains (Table 1).

**Table 1:** Total Sod activity of *Staphylococcus aureus *clinical isolates.

*S. aureus *strain	**Strain response to PDI**^**Δ**^	**Total Sod activity [U/mg of cell proteins]**^**1**^		**Total Sod activity [U/mg of cell proteins]**^**2**^		Sod activity increase [× fold]
			
		**Before PDI**^**3**^	**After PDI**^**3**^	**Before PDI**^**3**^	**After PDI**^**3**^	
	*MRSA*					
472	S	1494 ± 517	492 ± 96	16.7 ± 10.4	66.6 ± 5.8	3.9
2002	R	2006 ± 312	1247 ± 154	41.8 ± 6.5	43.3 ± 5.2	1.0
80/0	S	1604 ± 404	680 ± 93	24.6 ± 6.2	113.4 ± 15.5	4.6
4246	R	1703 ± 720	1807 ± 591	11.6 ± 4.9	34.4 ± 10.3	2.9
	*MSSA*					
1397	R	2234 ± 235	1046 ± 48	32.8 ± 3.4	28.5 ± 0.86	0.8
7259	R	1957 ± 805	1375 ± 178	46.6 ± 19.2	42.3 ± 5.3	0.9
2288	S	3596 ± 427	3583 ± 488	27.8 ± 3.3	137.2 ± 14.2	4.9
5491	S	2070 ± 318	2426 ± 42	25.2 ± 3.9	86.5 ± 1.5	3.4

^**1 **^- the given numbers are mean values of 3 measurements ± standard deviation, absolute values are given

^2 ^- values normalized with respect to the number of c.f.u. (colony forming units)

^3 - ^PDI - Photodynamic inactivation performed with 50 μM protoporphyrin IX, light dose of 12 J/cm^2^, 624 nm red light.

MRSA - Multiresistant *Staphylococcus aureus*; MSSA - Multisensitive *Staphylococcus aureus*

^**Δ **^S - sensitive, R - resistant

**Table 2:** Transcript level of the *sodA*, *sodM *genes in *Staphylococcus aureus *clinical isolates.

*S. aureus *strain	Strain response to PDI	***Sod *genes transcript level [copies/μl]**^**1**^		***Sod *genes transcript level [copies/μl]**^**2**^		Transcript level increase [× fold]
			
		**Before PDI**^**3**^	**After PDI**^**3**^	**Before PDI**^**3**^	**After PDI**^**3**^	
		*SodA*				
472	sensitive	372150	396674	418.1	5666.7	13.5
80/0	sensitive	1671	3136	2.5	52.2	20
1397	resistant	450267	24647	662.1	68.4	0.1
4246	resistant	4978943	1482683	3387.0	2745.7	0.8
		*SodM*				
472	sensitive	59205	194245	66.5	2774.9	41
80/0	sensitive	56789	21804	87.3	363.4	4.1
1397	resistant	123025	45475	279.6	119.6	0.4
4246	resistant	286623	198523	267.8	208.9	0.8

^1 ^- absolute number of amplified mRNA fragment

^2 ^- values normalized with respect to the number of c.f.u. (colony forming units)

^3 ^- PDI - Photodynamic inactivation performed with 50 μM protoporphyrin IX, light dose of 12 J/cm^2^, 624 nm red light.

### *sodA *and *sodM *gene transcript levels change after PDI

In order to assess if the increase in Sod activity takes place on the transcription level, we applied quantitative estimation of *sodA *and *sodM *gene transcript levels in several clinical *S. aureus *isolates. We chose two most susceptible PDI strains (472 and 80/0) and two most resistant to PDI treatment (1397 and 4246) in our experimental conditions. In both PDI-sensitive strains we observed an increase in the transcript level of both *sodA *and *sodM *genes. Particularly interesting seemed to be strain 472, in which the transcription level of *Sod *genes increased 13.5 times in the case of *sodA *gene and 41 times in the case of *sodM *gene (Table 2). In the second highly sensitive to PDI treatment, strain 80/0, the respective values of *sodA *gene transcription levels were also high and increased 20-fold, whereas in *sodM *a 4.1-fold gene transcription increase was observed (Table 2). On the contrary, in strains 1397 and 4246 we did not observe an increase in transcript levels of neither *sodA *nor *sodM *genes (Table 2). This result remains in good agreement with the total Sod activity rise after PDI treatment (Table 1).

**Table 3:** Primer sequence used for real-time PCR

Gene name^*^	Primer sequence (5'-3')	Amplification product size	Identification number of the gene
*sodA *for	TGC ACG CTT TGG TTC AGG TTG GG	177 b.p.	NCTC 8325 ID 3920105
*sodA *rev	GCG CCA ATG TAG TCA GGG CGT TTG		
*sodM *for	CCG GAA GCG ATG AGG ATG TCA GTC	132 b.p.	NCTC 8325 ID 3919804
*sodM *rev	TGC CCC ACT GCG CTT TGA TGT C		

* - for: forward primer, rev: reverse primer

## Discussion

*Staphylococcus aureus *is one of the most common human pathogens. It infects tissues locally, however through the action of a range of pyrogenic toxins and superantigens, bacteria can spread easily making the infection generalized [26]. The most dangerous therapeutically problematic are methicillin-resistant *Staphylococcus aureus *(MRSA), which are resistant not only to methicillin itself but also to all β-lactams as well as other groups of antimicrobial chemotherapeutics, like macrolides, lincosamides, aminoglycosides [27]. The latest epidemiological data indicates that the prevalence of MRSA in Europe seems to be low but is increasing, moreover European strains are very heterogeneous as opposed to USA-derived MRSA [28]. As the multiresistance spread is apparent among *S. aureus *strains in hospital settings, and becoming more evident in the community (so called community-aquired MRSA), several attempts are taken to develop strategies against these bacteria. The most popular and main-stream areas of research are new antimicrobial therapeutics [29]. However, alternative therapeutic options are also under investigation, to name: antimicrobial natural compounds [30,31], cationic antimicrobial peptides [32], the use of protection strategy to biofilm formation [33,34] or bacteriophage-based approaches [35,36]. Among the listed, photodynamic inactivation (PDI) of *S. aureus *is also a promising option. Photodynamic inactivation is based on a concept that a non toxic chemical, named a photosensitizer upon excitation with light of an appropriate wavelength is activated. As a consequence singlet oxygen and other reactive oxygen species are produced, which are responsible for the cytotoxic effect towards bacterial cells [37,38]. It is of great clinical importance and an advantage of PDI that *S. aureus *isolates, both MRSA and MSSA, can be effectively killed [39]. Previous reports of our group emphasized that *S. aureus *response to PDI is a strain-dependant phenomenon, which from the clinical point of view warrants attention [24]. Among 80 MRSA and MSSA strains some were ultra-sensitive to protoporphyrin IX diarginate-based PDI, whereas others exerted complete resistance to such treatment. The same tendency was observed in the presented results with the use of protoporphyrin IX as a photosensitizer (Figure 3). In our attempts to determine the molecular marker of strain-dependent response to PDI, we found that biofilm producing strains were killed less efficiently in comparison to non biofilm-producing strains [24], whereas efflux pumps, eg. NorA had no influence on the efficacy of photokilling [25].

### Sod status and PDI response

In the presented work we focused on the role of superoxide dismutases in the response of *S. aureus *to PDI. Superoxide dismutase constitutes the first line of bacterial defense against oxidative stress, therefore it was expected that the correlation may exist between the Sod status in the cell and response to PDI. Statistical analysis revealed no substantial difference in the survival rate among the four reference strains in TSB medium. In the study by Valderas and Hart, the same strains, deprived of either of the two Sods or both of them, were analyzed in conditions of methyl viologen (MV)-generated oxidative stress. They noticed that the highest drop in viability was observed in the case of SodAM double mutants grown in TSB medium [8]. On the contrary, the group of Foster, found that similar strains (i.e. analogues Sod mutants but with different genetic background) due to the action of internally-generated superoxide anion, viability drops in the case of both, SodA and SodAM double mutants in the Chelex treated BHI medium without Mn^++ ^ions. They also observed that upon supplementation of the medium with Mn^++ ^the viability of the mentioned mutants increased. When the same strains were challenged with externally generated superoxide anion in the stationary phase of growth, only the double Sod mutant was more susceptible to such treatment in comparison to the wild type SH1000 strain, moreover such an effect was not dependent on Mn^++ ^presence [16]. We performed statistical analysis of the data presented in Figure 1 and found no statistically relevant difference existing among the four strains analyzed (i.e. wild type RN6390, RN6390*sodA::tet*, RN6390*sodM::erm*, RN6390*sodM::erm sodA*::*tet*), what is seen in Figure 1. Our results differ from the one presented by Hart [8], which may be attributed to the differences in types of oxidative stress generated as a result of photodynamic action versus methyl viologen-induced oxidative stress used by Hart group. Methyl viologen is believed to induce internal oxidative stress. Our previous results showed that PDI-induced oxidative stress is mainly external [25]. In our previous work, when PpIX was washed away from the cell suspension before illumination, the photodynamic effect was abolished. Thus we can speculate that oxidative stress associated toxicity is a result of cell wall and bacterial membrane damage, which eventually leads to loss of cell viability. We can hypothesize that in our experimental conditions we used a more complex oxidative stress generating system than that used by Hart or Foster group. It is known that during photodynamic inactivation a number of reactive oxygen species are generated. This phenomenon is dependent on the type of photosensitizer used as well as medium conditions. For example, it was shown for fullerol c60, a recently studied photosensitizer, that depending on the medium used, either singlet oxygen alone or singlet oxygen together with superoxide anion were produced in a phototoxic process [40]. Different species of ROS produced in various media may affect the phototoxic effect on the same strain. We can speculate that apart from singlet oxygen and superoxide anion, other ROS can be generated in PpIX-mediated photodynamic process, which can affect either SodA or SodM regulatory pathways. The regulation of Sod activity in bacterial cells is very complex and yet not fully understood. Divalent metal ions, eg. Mn, Fe play a crucial role in these processes as enzyme or transcription factor regulator cofactors [16,41,42]. It is known that homeostasis of Mn and Fe are intertwined and most likely the manipulation of one of them greatly alters the uptake, storage and regulation of the other. It was shown that direct elemental superoxide scavenging by Mn occurs in *S. aureus *[12]. This effect was also clearly visible in our experimental data, where the survival rate of the double *S. aureus sodAM *mutant increased from 4.1 log_10 _units reduction in the Mn-depleted medium to 1.3 log_10 _units in the Mn-supplemented one (Figure 2) as a response to oxidative stress generating PDI. The comparison of the survival fraction of wild type RN6390 and *sod *mutants among each other as well as between conditions of Mn presence and absence in the medium explicitly indicates that Mn^++ ^ions influence the efficacy of bacteria killing but based on our results this seems to be regardless of the Sod activity.

### Clinical isolates of *Staphyloccocus aureus *diversely respond to PDI

Eight *S. aureus *strains isolated from hospitalized patients (4 MRSA and 4 MSSA) examined with respect of their ability to survive after PDI treatment, showed different pattern of response. Based on statistical analysis we divided those strains into two groups: sensitive and resistant to PDI. In the group of resistant strains (2002, 4246, 1397, 7259) the drop in the survival rate did not exceed 1.5 log_10 _units. In the second group of strains, called sensitive, (472, 80/0, 2288, 5491) the drop in survival rate was at least 1.5 log_10 _units reduction in viable counts. In our previous reports we already showed a strain-dependent response to PDI targeted *S. aureus *cells, where the observed efficacy of photokilling reached even 5 log_10 _units reduction. The differences between our previous studies and the one presented here might have probably resulted from a different photosensitizer used - PpIX vs. protoporphyrin IX diarginate (PpIXArg_2_) [24,25]. Other groups also observed the phenomenon of PDI-strain dependence, however, the mechanism underlying the diverse response to PDI was not explored [43,44]. Our data shows that at lower concentration of a photosensitizer (10 μM) a substantial drop in bacterial survival occurred, whereas at higher concentrations (25-50 μM), no further decrease in survival was noticed. We associate this phenomenon with poor solubility of PpIX in water solutions but the solubility itself does not justify the observed variability in killing curves. Similar results were obtained by the group of Wilson (2008). In the study they used another anionic photosensitizer, indocyanine green (ICG) against *S. aureus *and observed that the concentration of 25 μg/ml resulted in 6 log_10 _units reduction in viable counts, but higher ICG concentrations (50 and 100 μg/ml), resulted in lesser, about 4 and 5 log_10 _units reduction in survival counts, respectively [45]. Possible explanation of this phenomenon may be the self shielding effect of the non-bound PS in solution at higher concentrations. Effective photodynamic therapy is a result of a combination of several factors. Beside the biophysical properties of a sensitizer itself, also total light delivered, time of incubation with a photosensitizer, presence of additional proteins are crucial. In our work we did not focused on examining the dependence of killing rate vs. light dose. We performed all photodynamic inactivation studies on one light dose (12 J/cm^2^) chosen as optimal based on our previously published data concerning *S. aureus *photoinactivation as well as phototoxicity assays performed on dermal human fibroblasts [46,47].

In our previous attempts to explore the differences of porphyrin-based photokilling towards *S. aureus *cells, we found biofilm production ability to correlate with higher resistance to PDI treatment. However, it was also noted that among *S. aureus *isolates with elevated resistance to PDI, biofilm non-producing strains were also observed. This points out that biofilm production is not the only factor responsible for the observed phenotype [24]. Results published by Gad *et al*. indicated that extracellular slime significantly influences PS uptake by *S. aureus *cells, however an unambiguous conclusion was not possible due to the significant differences in both the uptake and PDI efficacy of the three PS tested, namely chlorine_*e6*_, poly-L-lysine-chlorine_*e6 *_and methylene blue [48]. *S. aureus *strains tested in our experimental conditions expressed no statistical correlation between PS uptake and PDI effectiveness, nevertheless the highest accumulation of PS was observed for the most efficiently killed strain 472 (3.4 log_10 _reduction in viable count units), as well as the lowest PS accumulation was observed in the case of the most resistant to PDI - strain 1397 (0.2 log_10 _reduction in viable count units) (Figure 3). The mean uptake level was 47.4 μg/mg of total protein content and 7.3 μg/mg of total protein content, for strains 472 and 1397, respectively. The results concerning uptake level in strains 472 and 1397 remain in a good agreement with our previous reports, where the same set of clinical isolates was analyzed but with the use of a different PS, namely PpIXArg_2 _[25]. Based on our previous and present results we conclude that the PS uptake process is not the main determinant of PDI effectiveness, at least for the porphyrin-based photokilling. We and other authors propose subsequent factors which may contribute and explain the differences in PDI efficacy of bacteria [25,49], eg. cellular repair systems or level of antioxidant enzymes.

### Sod activity and transcript level increase after PDI in PDI-susceptible strains

The participation of superoxide dismutase in oxidative stress resistance, and also in photodynamically generated reactive oxygen species is obvious. However, the role of Sod activity in PDI of bacteria has not been studied so far. There is few literature data on the association of Sod activity and photodynamic inactivation studies, and to the best of our knowledge they all concern eukaryotic cells. It was proposed for example that inhibition of Mn-Sod activity potentiates the antitumor effectiveness of photodynamic therapy in several cell lines and also in a mouse model of tumorigenesis [50]. Our attempt was to assess Sod activity in clinical isolates of *S. aureus *and to compare its basic level between PDI-resistant and PDI-susceptible bacteria. Basic Sod activity levels differed only slightly between PDI-resistant and PDI-susceptible strains (33.2 ± 15 U/mg and 23.6 ± 4 U/mg, respectively), which can be expected as *S. aureus *is not constantly exposed to elevated levels of oxidative stress After PDI treatment we observed about a 4-fold increase of Sod activity but only in strains susceptible to PDI. Sod expression is probably induced by a particular signal. The result published by the Foster group showed that when examining lacZ fusions with *SodA *genes, exposition of the cells to methyl viologen (internal oxidative stress generating agent) the level of SodA increased. The increase of SodM level was also observed, but only when cells were exposed to externally generated oxidative stress (xanthine/xanthine oxidase) [16]. Summarizing, although we did observe some differences of the basic Sod activity levels in PDI-susceptible vs. PDI-resistant strains, their statistical relevance is not obvious and does not explain the huge differences in PDI-based bactericidal efficacy (Table 2).

The reports previously published by our group showed that the bactericidal effect of PpIXArg_2_-based photokilling was almost completely abolished, when PS was washed away after incubation (before light exposure) [25]. This indicated that externally generated ROS are responsible for bacterial cell destruction. In regard to our currently presented results we also noticed that some amount of PS enters the cell and influences the transcription of certain genes, eg. *sodA *and *sodM*. We observed an increase in *sodA *and *sodM *transcript levels but only in 472 and 80/0, PDI-susceptible strains (Table 2). The strains recognized as PDI-resistant, namely 1397 and 2002, did not demonstrate higher *sodA *nor *sodM *transcript levels. These results correlate very well with Sod activity measurements observed in these strains.

However, Sod activity increase in only susceptible cells proves that this is probably not the only factor affecting *S. aureus *vulnerability to porphyrin-based PDI.

## Conclusions

We confirmed in the presented study that the protoporphyrin-based photokilling efficacy is a strain-dependent phenomenon. We showed that oxidative stress sensitivity caused by the lack of both Sod enzymes can be relieved in the presence of Mn ions and partially in the presence of Fe ions. The fact that Sod activity increase is observed only in PDI-susceptible cells emphasizes that this is probably not the only factor affecting *S. aureus *vulnerability to porphyrin-based PDI.

## Methods

### Light source

BioStimul Lamp which emits polarized (96% level of polarization) monochromatic light (624 nm ± 18 nm) (BIOTHERAPY, Czech Republic) was used for all irradiation experiments. The power of the lamp was measured using a light power meter (model LM1, CARL ZEISS, Jena, Germany). The delivered light energy was approx. 0.2 J/cm^2 ^per minute.

### Photosensitiser

Protoporphyrin IX (MP Biomedicals) stock solution was prepared in 100% dimethyl sulfoxide (DMSO) (Sigma-Aldrich) to the final concentration of 10 mM and kept in the dark at room temperature.

### Bacterial strains

In this investigation we used the reference *S. aureus *strains: RN6390, RN6390 *sodA*:: *tet *(lack of SodA activity), RN6390 *sodM*::*erm *(lack of SodM activity), RN6390 *sodM*::*erm sodA*:: *tet *(lack of SodA and SodM activities). These strains were obtained from the collection of Dr. Mark Hart from University of Arkansas, USA [8]. We also investigated eight *S. aureus *clinical strains isolated from patients from the Provincial Hospital in Gdansk, Poland. Among the clinical strains were four methicillin-sensitive *Staphylococcus aureus *(MSSA): 7259, 5491, 2288, 1397 and four methicillin-resistant *Staphylococcus aureus *(MRSA): 80/0, 2002, 4246, 472. The isolates were characterized by Gram-staining and their ability to produce coagulase and clumping factor using Slidex Staph Plus (BioMerieux). Additionally, the species were identified using the biochemical identification system ID 32 Staph (BioMerieux).

### Growth conditions

Strains were stored at 4°C on TSA plates (TSB containing 1.5% agar). For experimental purposes, a few colonies were inoculated into 5 ml of trypcase soy broth (TSB, BioMerieux) or Chelex-treated chemically defined metal limitation medium (CL) containing 400 μM MgSO_4 _and 1% glucose. Such broth cultures were grown overnight (18-24 h) at 37°C with rotation (250 rpm). After overnight growth, the optical density was adjusted to 0.055-0.06 at 600 nm, corresponding to approximately 1 × 10^7 ^colony forming units (c.f.u.) per ml. CL medium was prepared by adding 20 g Chelex-100 1^-1 ^and stirring at room temperature for 6 h prior the removal by filtration [41]. When needed 20 μM MnSO_4_, or FeSO_4 _was added to CL medium. Antibiotic-resistant *S. aureus *strains were maintained in the presence of either erythromycin or tetracycline (Fluka BioChemika) at the final antibiotic concentration of 5 μg/ml.

### Photodynamic inactivation studies

A photosensitizer solution, was added to 0.8 ml of the bacterial culture (OD_600 _= 0.055-0.06) to achieve the desired final concentration, from 10 to 50 μM. The culture was incubated at 37°C for 30 min. in the darkness and then loaded into a 96-well plate and irradiated. The total volume of the culture in each well was 0.1 ml. An identical microplate was incubated in the darkness in the same conditions and served as a control. After the illumination, aliquots (10 μl) were taken from each well to determine the number of colony-forming units (c.f.u.). The aliquots were serially diluted 10-fold in sterile phosphate buffered saline (PBS) to give dilutions from 10^-1 ^to 10^-4^. Aliquots (10 μl) of each of the dilutions were streaked horizontally on trypticase soy agar (TSA) (BioMerieux). After 18-24 h of incubation at 37°C in the darkness the formed colonies were counted and the results were analyzed statistically. There were three types of controls: bacteria untreated with photosensitizer (PS) and light, bacteria incubated with PS but kept in the darkness for the duration of the illumination, and bacteria exposed to light in the absence of PS. Each experiment was repeated three times. Decimal logarithm of c.f.u./ml was counted and normalized with respect to c.f.u./ml of control cells (untreated with PpIX). The results were shown as fractions of 1 in log_10 _scale.

### Preparation of cell lysates

Cell lysates were prepared from broth cultures of *S. aureus*. Cells were harvested by centrifugation (10,000 × g for 10 min at 4°C), washed with 1 ml of sterile PBS supplemented with 2 mM EDTA (ethylenediaminetetraacetic acid) and 1 mM PMSF (phenylmethylsulfonyl fluoride). Approximately 25 mg of glass beads (Sigma-Aldrich) were added to the cell suspension. The tubes were placed into a FastPrep (Bio 101) homogenizer and agitated at 6 m/s for 40 s. The lysates were cleared by centrifugation (12,000 × g, for 20 min at 4°C). The supernatant was recovered as 180 μl portions and stored at -20°C. Protein concentration was determined using the Bradford assay [51]. The experiment was repeated three times.

### SOD activity assay

The *S. aureus *clinical strains, during various phases of growth, were tested for SOD activity. Overnight (18-24 h) cultures were used to inoculate 5 ml of fresh TSB in 1:25 ratio. Cultures were incubated at 37°C with rotation (250 rpm). In order to assess Sod activity in cell extracts, samples were taken directly after PDI treatment. The proteins were extracted from lysate and the concentration was determined using Bradford assay [51]. The total SOD activity was determined by the inhibition of nitro blue tetrazolium (NBT) reduction [52], using 10 μl of protein sample per assay. The experiment was repeated three times.

### PpIX uptake studies

Overnight (18-24 h) cultures of *S. aureus *strains were inoculated to fresh TSB medium (OD_600 _= 0.3). One and a half ml of fresh bacteria suspensions were incubated in the dark at 37°C, 1 h with the final PpIX concentration of 10 μM or 50 μM. After incubation, the cell suspensions were centrifuged (1 min, 9000 rpm) and cells were washed twice with 1.5 ml of sterile PBS and centrifuged (1 min, 9000 rpm). Finally, the bacteria were lysed by digestion in 1 ml of 0.1 M NaOH-1% SDS (sodium dodecyl sulfate) for 24 h at room temperature to obtain a homogenous solution of the cell extracts. The fluorescence of the cell extracts was measured with a microplate reader (Victor, EG&G Wallac) in the amount of 0.1 ml per well. Separate fluorescence calibration curves were prepared with known amounts of PS dissolved in 0.1 M NaOH-1% SDS. The protein content of the entire cell extract was then determined by a modified Lowry method [51], using serum albumin dissolved in 0.1 M NaOH-1% SDS to construct calibration curve. Results were expressed as μg of PS per mg of cell protein [48].

### RNA extraction

Total RNA from PDI-treated cells was isolated directly after 60 min of illumination. Total RNA was isolated with the RNeasy Mini kit (QIAgen, Hamburg, Germany). *S. aureus *isolates were grown in 5 ml of tryptic soy broth (TSB) after 18 h of incubation with agitation at 37°C, (optical density OD_600 _= 2.0). Colony-forming units (c.f.u.) were measured by inoculating serial dilutions from the bacterial suspensions onto tryptic soy agar plates (TSA). A volume of 0.5 ml of the bacterial suspension was incubated with 1 ml of RNA Later™ (Ambion, Inc.) for 5 min. at room temperature. Cells were then centrifuged at 5000 rpm, 10 min. and the pellet was suspended in the commercial RTL buffer (QIAgen, Hamburg, Germany). About 25 mg of acid-washed glass beads were added to the solution. The tubes were placed into a FastPrep (Bio 101) homogenizer and agitated twice at 6 m/s for 40 s. with 1 min-interval on ice. The next steps were performed according to manufacturer's instructions. Finally, RNA samples were dissolved in 30 μl of RNase-free water. RNA integrity was tested with electrophoresis on 1% agarose gel. RNA quantification was performed measuring the absorbance at 260 nm. Nucleic acid purity was assessed measuring A_260_/A_280 _ratio (acceptable ratio was between 1.8 and 2.0).

### cDNA synthesis

Reverse transcription was performed with the use of commercially available QuantiTect Reverse Transcription kit (QIAgen, Hamburg, Germany). Firstly, 100 ng of total RNA was incubated with 2 μl of Wipeout buffer (QIAgen, Hamburg, Germany), containing RNase-free DNase, for 5 min. at 42°C. cDNA synthesis reaction was performed in a final volume of 20 μl, containing 100 ng of total RNA, 50 ng of random hexamer primers and the QuantiTect Reverse Transcriptase in RT buffer (QIAgen, Hamburg, Germany) according to the manufacturer's instructions for the first-strand cDNA synthesis.

### Quantitative real-time PCR conditions

The expression level of *sodA *and *sodM *genes were quantified using real-time RT-PCR (LightCycler^® ^FastStart DNA Master SYBR Green I; Roche Diagnostics). Two μl of cDNA were subjected to amplification in a 20-μl volume containing 5 μM concentration of each primer (Table 3), 3 mM of MgCl_2 _and 2 μl of ready-to-use Light Cycler^® ^DNA Master SYBR Green I (Roche Diagnostics). Pre-incubation step (95°C for 10 min.) was initially performed to activate FastStart DNA polymerase and to denature the template DNA. The following cycling conditions were used in the reaction: amplification and quantification program repeated 50 times (95°C for 5 s, 66°C for 15 s and 10 s extension at 72°C with a single fluorescence measurement), melting curve program (65-95°C with a heating rate of 0.2°C per second and a continuous fluorescence measurement) and finally a cooling step to 40°C. Specificity of the PCR products was confirmed by analysis of the dissociation curves.

Expression levels of *sodA *and *sodM *genes were measured using an absolute quantification method that allows to determine the exact copy concentration of target gene by relating the Ct value to a standard curve. Ct value is defined as the point at which the fluorescence rises appreciably above the background fluorescence. Standard curve was constructed by amplifying known amounts of target DNA. Standard curves for *sodA *and s*odM *genes were generated using serial dilutions of a standard sample (calibrator): 1×, 0.5×, 0.2×, 0.1×. As a calibrator, genomic DNA extracted from RN6390 strain (12.34 ng/μl) was used. In the case of *sodA *transcript quantification, amplification of *sodA *gene fragment was used, and similarly, to quantify *sodM *transcript level, *sodM *gene fragment from genomic DNA was used as calibrator. The quantitative data was generated based on different PCR kinetics of samples with different levels of target gene expression. The expression levels of *sodA *and *sodM *genes were compared to the data from a standard curve. The standard sample was included in every PCR run to control intra-assay variability.

### Statistical analysis

Each experiment was performed at least in triplicate. All primary data are presented as means with standard deviations of the mean. Statistical analysis was performed with one-way analysis of variance (ANOVA) with Tukey post-hoc test. Hypothesis were tested at significant level of 0.05. All analysis were performed using the STATISTICA version 8.0 software (StatSoft Inc. 2008, data analysis software system, Tulsa, USA).

## List of abbreviations

Sod: (superoxide dismutase); PpIX: (protoporphyrin IX); MRSA: (multi-resistant *Staphylococcus aureus*); MSSA: (multi-sensitive *Staphylococcus aureus*); ROS: (reactive oxygen species); PDI: (photodynamic inactivation); PS: (photosensitizer)

## Authors' contributions

JN: conceived the study, carried out the experimental work, analyzed the results and drafted the manuscript. EM: carried out experiments. MR: performed real-time PCR experiments. MG: provided technical support and helped to draft the manuscript. AGW: performed statistical analysis. KPB: helped to draft the manuscript. All authors read and approved the final manuscript.

## Supplementary Material

Additional file 1 **Fe ions influence on protoporphyrin IX-mediated PDI against reference strains**. The bacterial suspensions were illuminated after dark incubation for 30 min. at 37°C with different concentrations of PpIX (up to 50 μM). PDI was tested against reference strains of *S. aureus*: RN6390, RN6390*sodA*, RN6390*sodM*, RN6390*sodAM *in Fe-supplemented CL medium. Bacteria were illuminated with 12 J/cm^2 ^624 ± 18 nm light, and survival fractions were determined as described in Methods. Values are means of three separate experiments, and bars are SD. Click here for file
